# Differences in Intestinal Barrier Development between Intrauterine Growth Restricted and Normal Birth Weight Piglets

**DOI:** 10.3390/ani11040990

**Published:** 2021-04-01

**Authors:** Jarosław Olszewski, Romuald Zabielski, Tomasz Skrzypek, Piotr Matyba, Małgorzata Wierzbicka, Antoni Adamski, Elżbieta Grzesiuk, Maria Sady, Zdzisław Gajewski, Karolina Ferenc

**Affiliations:** 1 Veterinary Research Centre, Centre for Biomedicine Research, Centre for Regenerative Medicine, Department of Large Animal Diseases and Clinic, Institute for Veterinary Medicine, Warsaw University of Life Sciences, Nowoursynowska 100, 02-797 Warsaw, Poland; jarek.olszewski01@gmail.com (J.O.); rzabielski@icloud.com (R.Z.); pmatyba@vp.pl (P.M.); malgorzata-wielgosz@wp.pl (M.W.); msady92@gmail.com (M.S.); 2 Interdisciplinary Research Center, The John Paul II Catholic University of Lublin, 20-950 Lublin, Poland; dzikidzidz@wp.pl; 3 Department of Molecular Biology, Institute of Biochemistry and Biophysics, Polish Academy of Sciences, Pawińskiego 5a, 02-106 Warsaw, Poland; adamski.antoni1@gmail.com (A.A.); elag@ibb.waw.pl (E.G.)

**Keywords:** IUGR, intraepithelial leukocytes, goblet cells, Peyer’s patches, GALT

## Abstract

**Simple Summary:**

Animals with intrauterine growth restriction (IUGR) are defined as neonates born at term but with low birth weight and a characteristic shape of the head. A number of structural and functional modifications in the IUGR intestine affecting its digestive and absorptive function and impairing intestinal barrier function have been reported in the past. Far less is known about the immune system in the gut of IUGR pigs. Therefore, the aim of the present study was to evaluate the structures of the immune system of the gut mucosa in IUGR neonates. We found that the immune deficiency in the gut mucosa that results from restricted intrauterine development occurs at postnatal day (PD) 7, but it disappears thereafter within a week. However, all examined IUGR piglets had an increased number of intraepithelial leukocytes in the gut mucosa on PD 14. We have shown that the immune system of the gut of IUGR piglets is able to quickly compensate for the immunological deficiency postnatally and hardly shows any morphological disabilities in later life.

**Abstract:**

Intrauterine growth restricted (IUGR) piglets are born at term but have low birth mass and a characteristic shape of the head. Impaired general condition, especially in intestinal function, leads to an increase in the occurrence of diarrhoea and high mortality in the first days of life. So far, the mechanical and immunological gut barrier functions in IUGR are poorly understood. The aim of this study was to microscopically evaluate the early postnatal changes in the gut mucosa occurring in IUGR piglets. Whole-tissue small intestine samples were collected from littermate pairs (IUGR and normal) on postnatal day (PD) 7, 14 and 180 and analysed by light microscopy. We found that in the IUGR piglets, the percentage of intraepithelial leukocytes was reduced in the duodenum on PD 7, but it increased in the proximal and middle jejunum both on PD 7 and PD 14, which suggested the development of an inflammatory process. The number of goblet cells was also reduced on PD 14. The average size of the Peyer’s patches in the distal jejunum and ileum showed significant reduction on PD 7 as compared to normal pigs; however, on PD 14, it returned to normal. On PD 180, we did not find any differences in the measured parameters between the IUGR and the normal pigs. In conclusion, we found that in one-week-old IUGR pig neonates, the gut barrier and the immune system structures display signs of retarded development but recover within the second postnatal week of life.

## 1. Introduction

Intrauterine growth restriction (IUGR) is observed in 6% to 10% of all pig neonates [1]. One of the well-established consequences of IUGR in piglets is a high early postnatal mortality that reaches up to 75%. Most of the deaths in IUGR pig neonates occur in the first 2–3 days of life and are caused by crushing, due to reduced mobility, and by gastrointestinal and metabolic problems [1,2]. We have previously described a number of peculiarities in the development of the small intestine [3,4,5,6,7], pancreas [3], and liver [8] in IUGR pig neonates on histological and molecular levels. The main differences, compared to normal body weight (NBW) littermates, were observed in the gut mucosa (shorter and less numerous intestinal villi, changes in the crypt and villi mitosis to apoptosis ratio, reduced activity of brush border enzymes, and reduced secretion of gut hormones and growth factors). Microscopy studies also evidenced a marked delay in the disappearance of foetal-type enterocytes (FTEs) in the epithelium of the jejunum and ileum, as compared to normal pigs of the same age [1]. Moreover, we found that the microtubules and cisterns located in the upper part of the FTEs differed in IUGR piglets from those observed in NBW pig neonates, as well as the formation of large digestive vacuoles in the enterocytes of IUGR piglets [7]. All of these changes may directly impact on the digestion and absorption of nutrients in IUGR piglets [1], but may also be responsible for the decrease in the immunological status of the gut.

Studies showed that the development of the gut’s immune system and its responsiveness to allergens entering the small intestine may be different in IUGR piglets [9]. A temporary opening of the intestinal epithelial barrier is necessary to obtain passive immunity via the uptake of colostrum immunoglobulins, but a prolonged presence of foetal enterocytes facilitates the penetration of the intestinal epithelium by pathogens. Dong et al. showed that the function of the gut-associated lymphoid tissue (GALT) is significantly impaired in IUGR animals compared to newborns with normal birth weight [8]. They also found a smaller number of epithelial goblet cells and lymphocytes in the small intestinal epithelium of IUGR neonates. D’Inca and co-workers [10] showed an increased number of bacteria adhering to the intestinal mucosa in IUGR piglets compared to normal piglets. These results were corroborated by an increase in the expression of the genes encoding the proteins responsible for bacterial adherence. They postulated that a defence mechanism against excessive bacterial colonization in IUGR piglets may stimulate the expression of genes responsible for mucin secretion in the mucus-producing goblet cells [10]. More recent studies report differences in the methylation of 33 genes in the small intestines of IUGR piglets compared to normal piglets, which could lead to future metabolic dysfunction (concerning carbohydrate, lipid, and protein metabolism) and immune response differences [11,12]. Moreover, mRNA and miRNA studies indicated increased intestinal barrier permeability due to changes in tight junction development in IUGR piglets [13,14].

Though the differences in the structure of the intestinal mucosa between IUGR and the NBW piglets have been studied, little is known about the development of the overall gut barrier function in suckling IUGR piglets. Therefore, the aim of this study was to investigate the morphological and immunological barriers in the gut mucosa of IUGR piglets at an early postnatal and adult age.

## 2. Materials and Methods

### 2.1. Animals and Tissue Collection

This protocol was conducted in compliance with the European Union regulations concerning the protection of experimental animals. The study protocol was approved by the Local Ethical Committee, Warsaw University of Life Sciences, Warsaw, Poland (No: 37/2015; WAW2/046/2018). The study was performed in a pig farm with 650 purebred sows (Polish Landrace). Sows delivered between 9 and 12 piglets, which resulted in 28 neonates from one sow per year. The health status of the animals was high due to high farm sanitary and epizootic standards. Farrowing, growing, and fattening sectors had slatted floors and controlled ventilation, temperature, and humidity. Sows were kept on a standard diet during pregnancy (dry matter (DM) 87.6%, metabolizable energy (ME) 11.35 MJ/kg, and crude protein (CP) 13.1%) and lactation (DM 87.3%, ME 12.93 MJ/kg, and CP 15.4%). Fresh food and water were provided daily ad libitum. On postnatal day (PD) 3, all piglets were injected intramuscularly with 100 mg of iron dextran (FeDex, Ferran100, 10% solution, Vet-Agro, Lublin, Poland). Suckling piglets were creep fed from the third week of life and weaned on postnatal day 28. After weaning, pigs were fed ad libitum using a commercial starter, grower, and finisher diet.

The newborn piglets from multiparous sows were checked for body weight at birth. All neonates of low birth weight (0.6–0.9 kg) with the distinctive facial shape characteristic of IUGR neonates [15,16] were marked. IUGR piglets were kept together with their litter, fed by the sow, and weaned on postnatal day 28. On PD 7, seven clinically healthy piglets recognized at birth as IUGR were killed by barbiturate overdose for sampling of the small intestine–duodenum (50% of the length); proximal, mid, and distal jejunum (respectively, 25%, 50%, and 75% of the length); and ileum (50% of the length). The control group (NBW) consisted of 7 clinically healthy piglets selected on PD 7 from the same litters as the IUGRs. The controls were piglets of normal birth weight (NBW, between 1.3 and 1.6 kg) that represented the average birthweight of all its littermates. The same protocol was repeated on postnatal day 14 and 180 to obtain another two sets of IUGR–NBW pairs of animals for histology study.

### 2.2. Histological Staining

Whole tissue intestinal samples were fixed using a tissue processor (Leica TP1020, Kawa.ska, Zalesie Górne, Poland) (dehydration in increasing concentrations of ethanol, xylene washing, and paraffin embedding). Samples were dissected into 5 μm sections (microtome Leica RM2255, Kawa.ska, Poland) and processed using the standard haematoxylin and eosin staining protocol (Multistainer Leica ST5020, Kawa.ska, Poland).

### 2.3. Optical Microscopy Analysis

Using an optical microscope (Olympus BX43) equipped with a digital camera and the CellSens v.3 (Olympus, Tokyo, Japan) software, the perimeters and areas of Peyer’s patches in the distal jejunum and ileum were measured with the 10× objective for neonatal (PD 7 and 14), and the 4× objective for adult (PD 180) pigs. Only complete sections of Peyer’s patches were chosen for analysis (both the basal round-shaped part and the top part had to be intact and free of artefacts). For the measurement of the sum of the areas of Peyer’s patches, all Peyer’s patches (also partial sections) were measured to provide the area of lymphoid tissue in the section. The cell number counts in the Peyer’s patches were performed using the MicroImage (Olympus) software.

The percentage of foetal-type enterocytes [5] and goblet cells in the epithelium and the percentage of intraepithelial leukocytes was measured as follows. Firstly, the epithelial lineage area was discriminated on the villus using the area of interest option (MicroImage, Metro Manila, Philippines). Secondly, the total number of cell nuclei was measured in the marked area of interest (MicroImage). Thirdly, the enterocytes containing large vacuoles (i.e., the foetal-type enterocytes), goblet cells, and intraepithelial leukocytes were counted manually in the area of interest. This procedure was repeated on 6 to 10 villi in one slide, and finally, the percentage of foetal-type enterocytes, goblet cells, and intraepithelial leukocytes to all epithelial cells was calculated. For each slide, the villi were chosen for cell counting based on their structural integrity, profile shape, size, and morphological consistency. For each intestinal segment, at least 5 slides were analysed.

### 2.4. Scanning Electron Microscopy

Formaldehyde-fixed sections were washed in saline and dehydrated in a series of alcohol solutions. After drying in a critical point drier, samples were sputter coated with a layer of gold–palladium (Au/Pd) and examined using an Ultra Plus (Zeiss) SEM. For a complete description of the method, refer to Skrzypek et al. [5,6,7].

### 2.5. Statistical Analysis

The results were subjected to two-stage statistical analysis. Normal distribution was assessed using the Kolmogorov–Smirnov test. Equality of the variance was performed using the F test for unpaired *t*-tests. Accordingly, for one-way ANOVA, Bartlett’s test was performed. Depending on the outcome, *t*-test data were then analysed with an unpaired *t*-test or Welch’s *t*-test (in the absence of normal distribution) to evaluate the differences between NBW and IUGR animals. For all parameters between the time points (PD 7, 14, and 180), one-way ANOVA followed by Tukey’s post-test was used. All statistical analyses were done using GraphPad Prism v.5.0 (GraphPad Software, San Diego, CA, USA); *p* < 0.05 was considered significant, *p* < 0.01 highly significant, and *p* < 0.1 a trend.

## 3. Results

### 3.1. Pig Body Weight

The body weight of the NBW and IUGR pigs is given in Table 1. At each time point, the body weight of the IUGR pigs was significantly lower compared to the NBW animals. During the first two weeks of life, the body weight of IUGR piglets was only 54–61% of the control (NBW) piglets; however, on PD 180, the body weight of IUGR pigs was about 91% of the controls.

### 3.2. Disappearance of Foetal-Type Type Enterocytes as a Marker of Intestinal Mucosa Development

**Figure 1:**
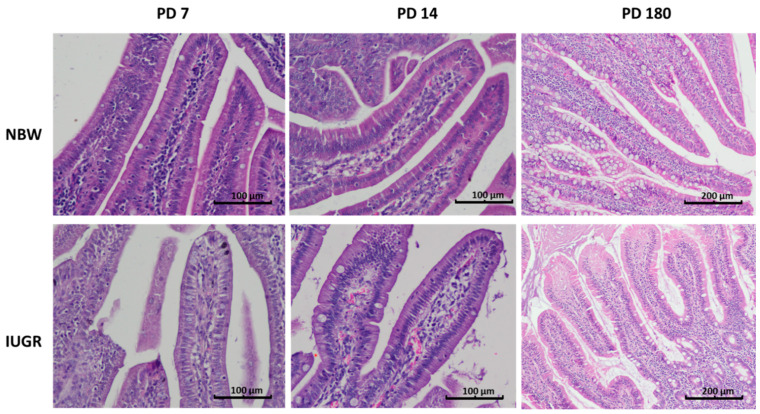
Duodenal villi in normal birth body weight (NBW—top panel) and intrauterine growth restricted (IUGR—bottom panel) pigs on postnatal day (PD) 7, 14, and 180. On PD 7, there were still visible single foetal-type enterocytes containing large vacuoles, as well as a small number of intraepithelial leukocytes in the IUGR piglets compared to the NBW piglets. On PD 14, the number of goblet cells in the IUGR animals was lower than in the NBW animals. Haematoxylin and eosin staining; objective: 10× for PD 7 and PD 14, and 4× for PD 180.

**Figure 2:**
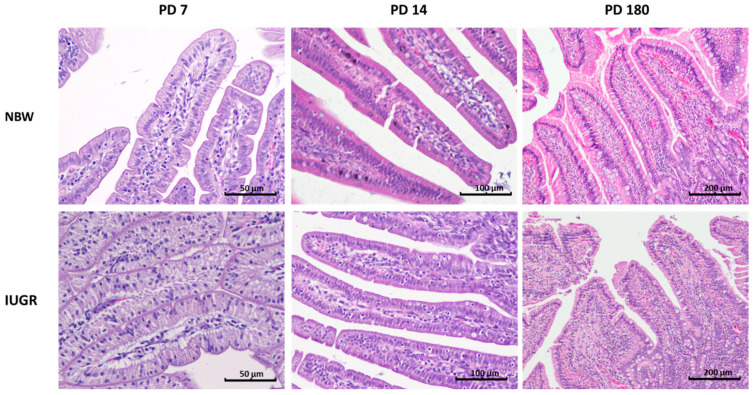
Mid-jejunum villi in normal birth body weight (NBW—top panel) and intrauterine growth restricted (IUGR—bottom panel) pigs on postnatal day (PD) 7, 14 and 180. On PD 7, the Figure 1. of the upper part of the villi in the IUGR piglets, whereas in their NBW littermates, foetal-type enterocytes were very rare. In this segment of the gut, abundant intraepithelial leukocytes were observed in IUGR individuals both on PD 7 and PD 14. Analyses also revealed a decrease in the percentage of goblet cells on PD 14. Haematoxylin and eosin staining; objective: 10× for PD 7 and PD 14, and 4× for PD 180.

**Figure 3:**
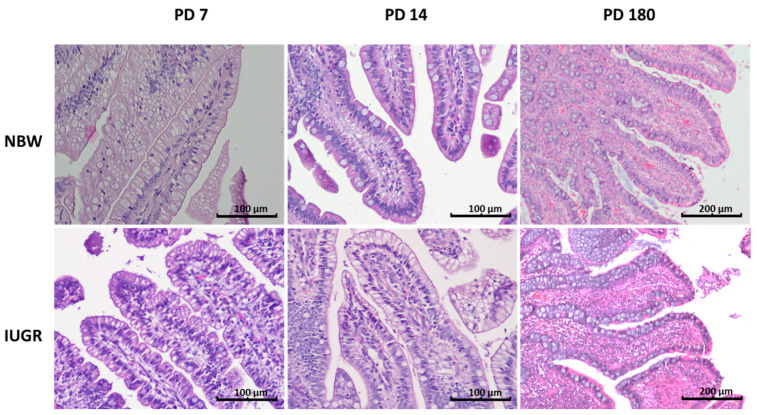
Ileal villi in normal birth body weight (NBW—top panel) and intrauterine growth restricted (IUGR—bottom panel) pigs on postnatal day (PD) 7, 14, and 180. On **PD** 7, foetal-type enterocytes were commonly seen in both IUGR and NBW pig neonates, but the percentage of foetal-type enterocytes was much higher in the IUGR piglets compared to the NBW piglets. On PD 14, the rebuilding of the mucosa, consisting of the replacement of the foetal-type enterocytes with enterocytes that lack the large-sized vacuoles, was nearly complete in the NBW but not in the IUGR neonates, in which there were still plenty of vacuolated enterocytes in the epithelium. No differences were found between the NBW and the IUGR animals with respect to the percentage of intraepithelial leukocytes and goblets cells. The percentage of intraepithelial leukocytes was, however, much higher on PD 180 than in the neonates. Haematoxylin and eosin staining; objective: 10× for PD 7 and PD 14, and 4× for PD 180.

One of the important markers of gut mucosa development in pig neonates is the disappearance of foetal-type enterocytes with age (Table 2). The NBW mucosa showed normal dynamics of the changes occurring within the first two postnatal weeks, namely a gradient from duodenum to ileum and a reduction of the percentage of the FTE in each examined intestinal segment with age. In contrast, the intestinal mucosa of the IUGR neonates showed a slower reduction in FTE in all segments of the small intestine, both on PD 7 and PD 14. This suggests that the remodelling of the gut epithelium in IUGR neonates is delayed (Table 2, Figure 1, Figure 2 and Figure 3).

### 3.3. Intraepithelial Leukocytes

In the NBW pig neonates, the percentage of intraepithelial leukocytes (IEL) was lower than in the six-month-old pigs (PD 180); this difference was evident in each of the examined segments of the small intestine (Table 3). Moreover, in the NBW neonates, the highest number of IELs was found in the duodenum and in the terminal small intestine (distal jejunum and ileum) (Figure 2), whereas in adult pigs, no such pattern was observed. In the IUGR neonates, the above-mentioned IEL pattern along the gut was not observed, and there were many spatial–temporal differences in the IEL number compared to their NBW littermates (Table 3, Figure 1, Figure 2 and Figure 3). In adults, there were no differences between the NBW and IUGR animals in regard to the IEL number.

### 3.4. Goblet Cells

The proportion of goblet cells in the epithelium increased along the small intestine from approximately 6% in the duodenum up to approximately 20% in the ileum (Table 4).

This pattern was observed in normal animals as well as in their IUGR littermates, though the sharpest differences over time were observed in the distal jejunum. On postnatal day 14, the number of goblet cells declined in the IUGR piglets in comparison to their NBW littermates, but this pattern did not continue with time (Table 4, Figure 1, Figure 2 and Figure 3).

### 3.5. Peyer’s Patches

**Figure 4:**
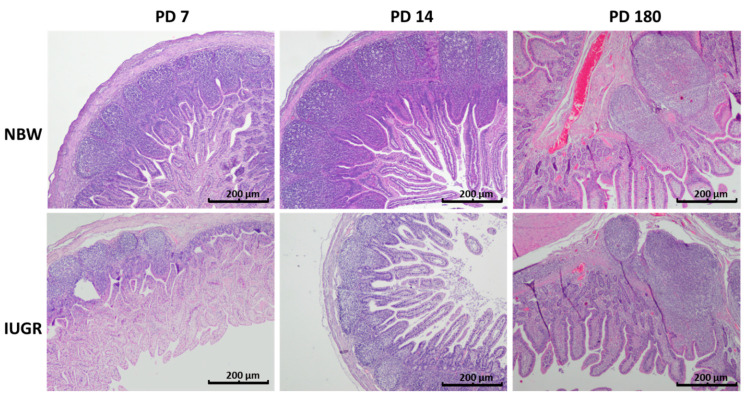
Peyer’s patches in the distal jejunum of normal body weight (NBW) and intrauterine growth restricted (IUGR) pigs on postnatal days 7, 14, and 180 (PD 7, PD 14, and PD 180). On PD 7 and PD 14, the average area of the Peyer’s patch in the IUGR individuals was significantly smaller than in their NBW littermates. Moreover, on PD 14, the number of cells per Peyer’s patch was decreased. Haematoxylin and eosin staining; objective: 4×.

**Figure 5:**
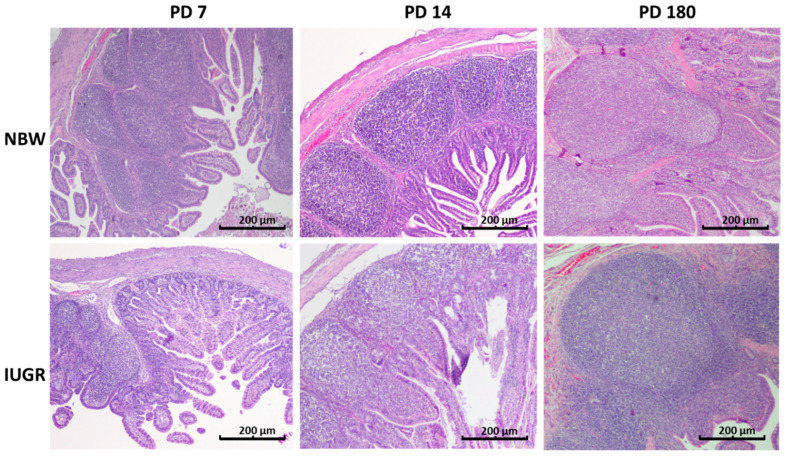
Peyer’s patches in the ileum of normal body weight (NBW) and intrauterine growth restricted (IUGR) pigs on the postnatal day 7, 14, and 180 (PD 7, PD 14 and PD 180). On PD 7, the average area of the Peyer’s patches and the density of cells per Peyer’s patch were significantly smaller in the IUGR piglets than in their NBW littermates. One week later, however (PD 14), the differences between the IUGR and the NBW samples were no longer observed, nor were any differences in the structure of the Peyer’s patches observed on PD 180. Haematoxylin and eosin staining; objective: 4×.

**Figure 6:**
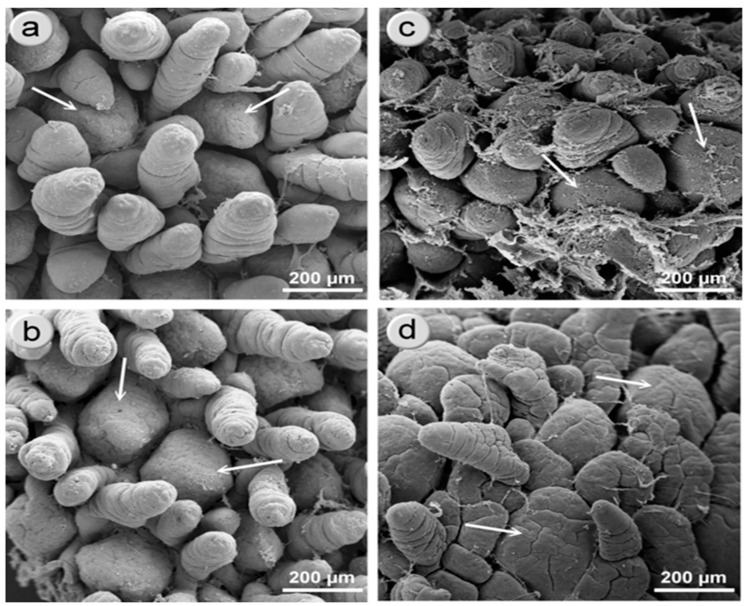
Scanning electron microscopy microphotographs of the surface of the iliac mucosa on postnatal day 7 (**a**—NBW, **c**—IUGR) and postnatal day 14 (**b**—NBW, **d**—IUGR). The microphotographs show the ileal villi and the Peyer’s patches (white arrows). The villi of the iliac mucosa of the NBW piglets were longer and finger-like. The villi of the IUGR piglets have different, leaf-like shapes on both postnatal day (PD) 7 and 14. At the apices of the villi of the NBW pigs at PD 14, there were clearly visible zones exhibiting shedding activity. The Peyer’s patches of the IUGR pigs were less developed than those of the NBW pigs on PD 7, but not on PD 14. On PD 7 and PD 14, the observed Peyer’s patch surfaces were flat, without the characteristic domed shape. This was also seen in the haematoxylin and eosin sections.

On PD 7 and PD 14, significant differences in the average area of Peyer’s patches were found in the distal jejunum of normal and IUGR piglets (Figure 4). Additionally, on PD 14, the average number of cells per Peyer’s patch (PP) was lower in the IUGR animals than in the NBW animals (Table 5). In the ileum, a significant decrease in the number of cells per PP, the average area of PPs, and the total area of PPs were found in the IUGR animals on PD 7. Interestingly, all differences were gone one week later (Table 5, Figure 5). Scanning electron microscopy revealed differences in the shape of the Peyer’s patches between the normal and IUGR neonates (namely, the upper surface of the Peyer’s patches of the IUGR animals was flat in contrast to its characteristic domed shape seen in the normal neonates) (Figure 6). Similar findings were obtained in the 2D histology slides (Figure 5). Both in the IUGR pigs and in their normal littermates, the size, total area of PPs per section, and the number of cells per PPs were strongly linked to the neonatal period. On PD 180, no significant differences in the structure or the area size of the ileal PPs were observed between the IUGR and the normal pigs (Table 5).

## 4. Discussion

In our previous studies, we found several important alterations in the intestines of IUGR pig neonates that could directly affect the structure and function of the intestinal barrier. These include the protracted appearance of foetal-type (vacuolated) enterocytes, decreased height of the villi and reduced depth of intestinal crypts, reduced rate of enterocyte apoptosis in the apical parts of the villi, and acceleration of apoptosis in the crypt region, as well as molecular changes in the enterocytes affecting their metabolism [1,3,4,5,6,7]. Other studies also suggested that the immune barrier in the intestines of IUGR pigs could be impaired at a different stage of their postnatal life [9,17,18,19]. In the present study, we have found that in the first one to two weeks of life, the mechanical barrier and the immune system of the small intestine of IUGR piglets was weaker than those of their normal body weight (NBW) littermates. Namely, the dynamics of the replacement of the FTEs with the adult-type enterocytes was slower in IUGR individuals than in NBW individuals, and thus more macromolecular components and microorganisms could potentially cross the epithelial barrier. Moreover, in the distal jejunum and ileum of the IUGR animals, the enterocytes formed several smaller vacuoles instead of one large vacuole, reducing the effectiveness of the lysosomal enzymes in digesting the content of the vacuoles [7]. This peculiarity, along with the differences in the expression of brush border enzymes and transport proteins [3,4], may negatively affect the absorption of nutrients. The present results corroborate our previous findings that foetal-type enterocytes may persist in IUGR piglets until postnatal day 28 [4], thereby softening the integrity of the intestinal barrier for a much longer time than what is the physiological window in normal neonates.

In the seven-day-old IUGR piglets, the number of intraepithelial leukocytes in the duodenum was significantly lower than in their normal littermates, which suggests a slower physiological migration of the leukocytes to the epithelium. Dong et al. [9] also found a reduced number of intraepithelial leukocytes in the small intestine just after birth, but in the ileum, not in the duodenum and the jejunum. They also noticed a decreased level of tumour necrosis factor-α (TNF-α) and interferon-γ (IFN-γ) in the jejunum and the ileum of IUGR piglets. Moreover, Wang et al. [19] noted that the levels of blood inflammatory cytokines and immunoglobulins G (IgGs) were lower at birth in IUGR piglets than in their normal littermates, suggesting that the overall activity of the immune system is also lower in IUGR animals than in normal piglets. On the other hand, we have found a markedly increased number of intraepithelial leukocytes in the proximal and mid jejunum of the IUGR neonates (on PD 7 and PD 14). This increase was, however, not sufficient to meet the criteria for inflammation, as no other signs of inflammation were observed microscopically, and the post-mortem analysis did not show any clinical signs of inflammation in the gut mucosa. Inflammatory reactions were previously observed both in the blood and in the intestines of IUGR piglets at early neonatal stages, at 12 h after birth, and at PD 5 [10,20]. In this context, our results may suggest that proinflammatory processes are more common in the jejunum of IUGR pig neonates than in their normal littermates. Correspondingly, Amdi et al. showed an increase in neutrophil percentage but a decrease in CD4+ T-cells and a lower level of LPS-induced IL-1β production in the blood of IUGR pigs, suggesting immunological hypo-responsiveness at this stage [21]. However, in adult pigs, our microscopy studies did not show any differences in the immune components of the gut barrier between IUGR and normal pigs. It is possible that the differences were compensated at the level of the gut mucosa but not in the circulating blood. Alternatively, it is possible that while the gut barrier structure looks normal, certain dysfunctions might be observed on a molecular level, including, e.g., the kinetics of cytokine production. Moreover, from the above-mentioned studies as well as from our study, it is not clear what may happen after weaning—more studies are necessary.

A decreased percentage of goblet cells was clearly observed in the IUGR neonates but only in the upper part of the small intestine (in the duodenum and the proximal and middle jejunum). Others have found a decreased number of goblet cells only at birth [9]. Another interesting observation is that the percentage of goblet cells did not increase in the IUGR animals between postnatal day 7 and 14, as is the case in their normal littermates, which further confirms that the development of the gut mucosa is delayed in the IUGR piglets. As recently suggested by Zhang and co-workers [22], this delay probably ends after weaning. The decreased percentage of goblet cells in the neonatal period leads to a decreased secretion of mucins, the major product of goblet cells, thereby enhancing the likelihood of an increase in bacterial adhesion to the intestinal epithelium [10,20,22]. Huang et al. found that 12 h after birth, IUGR and normal neonates already display distinct differences in bacterial colonization, which, along with the delayed maturation of enterocytes, may constitute an early factor promoting enteritis in IUGR animals [20].

The area of the Peyer’s patches increases steadily from birth to adulthood [23], though some declines in size may occur periodically due to stress, such as at weaning [24]. Our results also show intense enlargement of the areas of Peyer’s patches both in the IUGR animals and in normal piglets. At postnatal day 7, the average area and the sum of areas of the Peyer’s patches, as well as the average cell density per Peyer’s patch, in the distal small intestine were smaller in the IUGR animals than in their normal littermates. Surprisingly, within just one environment week, all measured Peyer’s patch parameters in the IUGR neonates increased to match the numbers found in normal piglets. Nevertheless, the differences in the shapes of the Peyer’s patches in the IUGR animals still remained. Accordingly, Stokes at al. [25] observed that the immunological system of the pig intestines grows rapidly in the first two postnatal weeks, and that after that time, it differentiates to achieve its “adult architecture” [25,26]. Our results show that the dynamics of the development of Peyer’s patches in the IUGR animals in the first two weeks of life is even faster than in normal piglets, which is contrary to the kinetics of enterocyte remodelling (which can take even longer than four weeks) [4]. On the one hand, this may be the result of the powerful immunostimulatory effect of environmental bacteria (pathogens) [27,28]. On the other hand, the immune system development observed in the guts of IUGR piglets in our research may be attributable to the fact that all piglets received similar portions of colostrum and milk from the sows. Our hypothesis assumes that in our study, the examined litters were of rather small sizes (from 9 to 12 piglets), so the competition to access colostrum and milk was not high, which, as shown by Amdi et al. [15], may be a crucial factor for maintaining good performance of IUGR piglets. Moreover, weaning piglets very early (i.e., prior to a period of intensive gut development (before PD 14)) had a significant negative effect on the size of the Peyer’s patches, which did not revert before or after weaning [23]. In contrast, in larger litters, even normal piglets suffered from immune deficiency due to inadequate intake of colostrum [29]. This very intense growth of the immune system in the lower gut of IUGR animals in the second week of life might be one reason, unforeseen so far, for the poor daily gain in body weight, since the immune system of the gut is very demanding with respect to the supply of energy and amino acids [30,31]. Accordingly, the catch-up growth of the IUGR neonates starts only after the phase of intense immune system growth (around weaning in our study) and thereafter. Another interesting point to study would be to clarify whether this fast growth of the Peyer’s patches somehow affects the populations of mucosal leukocytes and their secretory functions, including the release of cytokines and antibodies. It would be worth analysing this separately for the distal and the ileal Peyer’s patches, since Maroilley et al. [32] found some differences in their composition. More research in this area is needed. Table 6 gives a brief summary regarding our knowledge on the development of the immune barrier in IUGR piglets.

## 5. Conclusions

We have found that the immune system development in the lower gut (distal jejunum and ileum) of intrauterine growth restricted piglets makes great compensatory progress between postnatal days 7 and 14. During this period, some defects in the morphological structure of the immune system disappear. Nevertheless, the decreased percentage of goblet cells and the increased level of intraepithelial leukocytes may ease the tendency of the neonates to develop enteritis. More studies are necessary in this area in order to better understand the range and the kinetics of this development.

## Figures and Tables

**Table 1:** Body weight of the normal body weight (NBW) and intrauterine growth restricted (IUGR) Polish Landrace pigs (mean ± standard deviation (SD), *n* = 7).

Age (Days)	NBW (kg)	IUGR (kg)	*p*-Value
At birth	1.58 ± 0.27	0.86 ± 0.12	0.001
7	2.88 ± 0.52	1.64 ± 0.30	0.001
14	5.94 ± 1.03	3.64 ± 0.91	0.001
180	124.0 ± 3.3	114.9 ± 5.4	0.01

The *p*-values for the IUGR and NBW pairs were analysed using a *t*-test.

**Table 2:** Percentage of the foetal-type enterocytes in the small intestine at postnatal day (PD) 7 and PD 14 in normal birth body weight (NBW) and intrauterine growth restricted (IUGR) piglets (mean ± SD, *n* = 7).

Percentage of Foetal-Type Enterocytes (%)			
		PD 7	PD 14
Duodenum	NBW	Absent	Absent
	IUGR	2.12 ± 1.3	Absent
	*p-*Value	-	-
Proximal jejunum	NBW	Absent	Absent
	IUGR	14.02 ± 1.32	Absent
	*p-*Value	-	-
Middle jejunum	NBW	Very rare	Absent
	IUGR	33.4 ± 12.4 ^a^	1.7 ± 1.53 ^b^
	*p-*Value	0.01	-
Distal jejunum	NBW	34.1 ± 14.6 ^a^	12.57 ± 3.52 ^b^
	IUGR	67.2 ± 30.8 ^a^	35.1 ± 22.67 ^b^
	*p-*Value	0.39	0.37
Ileum	NBW	24.1 ± 8.61 ^a^	5.54 ± 1.34 ^b^
	IUGR	53.55 ± 21.83 ^a^	18.9 ± 6.81 ^b^
	*p-*Value	0.01	0.001

The *p*-values for the IUGR and NBW pairs were obtained using *t*-tests. ^a,b^ Different letters in superscript indicate significant differences between PD 7 and PD 14 in each row (*p* < 0.05).

**Table 3:** Percentage of intraepithelial leukocytes in the small intestines of 7-, 14- and 180-day-old (PD 7, PD 14 and PD 180) normal birth body weight (NBW) and intrauterine growth restricted (IUGR) pigs (mean ± SD, *n* = 7).

Percentage of Intraepithelial Leukocytes (%)				
		PD 7	PD 14	PD 180
Duodenum	NBW	15.29 ± 2.45 ^a^	12.30 ± 2.18 ^b^	21.3 ± 2.88 ^c^
	IUGR	8.87 ± 2.44 ^a^	13.44 ± 2.42 ^b^	23.06 ± 5.53 ^c^
	*p-*Value	0.0001	NS	NS
Proximal jejunum	NBW	2.74 ± 0.36 ^a^	5.26 ± 0.92 ^b^	39.7 ± 10.12 ^c^
	IUGR	4.02 ± 1.32 ^a^	7.47 ± 0.80 ^b^	36.19 ± 3.62 ^c^
	*p-*Value	0.03	0.009	NS
Middle jejunum	NBW	7.0 ± 1.92 ^a^	9.36 ± 1.41 ^b^	20.48 ± 4.00 ^c^
	IUGR	13.04 ± 2.17 ^a^	11.54 ± 1.32 ^a^	18.36 ± 4.54 ^b^
	*p-*Value	0.0003	0.02	NS
Distal jejunum	NBW	11.89 ± 3.70 ^a^	12.57 ± 3.52 ^a^	26.6 ± 6.29 ^b^
	IUGR	10.74 ± 2.58 ^a^	11.10 ± 2.67 ^a^	28.5 ± 2.52 ^b^
	*p-*Value	NS	NS	NS
Ileum	NBW	6.96 ± 1.49 ^a^	10.54 ± 1.75 ^a^	26.2 ± 6.96 ^b^
	IUGR	5.55 ± 1.83 ^a^	9.16 ± 2.19 ^a^	27.8 ± 9.65 ^b^
	*p-*Value	NS	NS	NS

The *p*-values for IUGR and NBW pairs were obtained using *t*-tests; NS—not significant; ^a,b,c^ different letters in superscript indicate significant differences in each row (*p* < 0.05; one-way ANOVA followed by Tukey’s post-hoc test).

**Table 4:** Percentage of goblet cells in the small intestines of 7-, 14-, and 180-day-old (PD 7, PD 14 and PD 180) normal birth body weight (NBW) and their intrauterine growth restricted (IUGR) pig littermates (mean ± SD, *n* = 7).

Percentage of Goblet Cells (%)				
		PD 7	PD 14	PD 180
Duodenum	NBW	6.67 ± 1.86 ^a^	10.81 ± 2.62 ^b^	7.71 ± 3.46 ^a,b^
	IUGR	5.52 ± 1.31	6.44 ± 0.68	6.74 ± 1.81
	*p-*Value	NS	0.0005	NS
Proximal jejunum	NBW	7.27 ± 1.69	9.10 ± 1.66	9.7 ± 3.08
	IUGR	5.85 ± 0.98	5.95 ± 1.06	6.38 ± 2.57
	*p-*Value	NS	0.005	NS
Middle jejunum	NBW	5.95 ± 1.39 ^a^	8.88 ± 1.62 ^b^	9.66 ± 2.14 ^b^
	IUGR	4.93 ± 1.34 ^a^	6.53 ± 1.3 ^b^	8.05 ± 3.09 ^b^
	*p-*Value	NS	0.02	NS
Distal jejunum	NBW	9.29 ± 2.62 ^a^	10.74 ± 2.58 ^b^	17.0 ± 2.81 ^c^
	IUGR	8.74 ± 2.25 ^a^	8.26 ± 2.17 ^a^	15.6 ± 5.47 ^b^
	*p-*Value	NS	NS	NS
Ileum	NBW	22.4 ± 3.49	20.54 ± 4.61	23.6 ± 6.08
	IUGR	20.15 ± 3.53	19.16 ± 3.03	18.2 ± 7.10
	*p-*Value	NS	NS	NS

The *p*-values for IUGR and NBW pairs were obtained using *t*-tests; NS—not significant; ^a,b,c^ different letters in superscript indicate significant differences in each row (*p* < 0.05; one-way ANOVA followed by Tukey’s post-hoc test).

**Table 5:** Histometric analysis of intestinal Peyer’s patches in normal birth body weight (NBW) and intrauterine growth restricted (IUGR) animals on the postnatal day 7, 14, and 180 (PD 7, PD 14 and PD 180) (mean ± SD, *n* = 7).

		Distal Jejunum			Ileum		
		PD 7	PD 14	PD 180	PD 7	PD 14	PD 180
Average number of cells per Peyer’s patch	NBW	1450 ± 614	1554 ± 454	N/A	1203 ± 204 ^a^	1620 ± 230 ^b^	6020 ± 2028 ^c^
	IUGR	1116 ± 538	1003 ± 131	N/A	601 ± 173 ^a^	1340 ± 347 ^b^	5179 ± 1392 ^c^
	*p-*Value	NS	0.0261		0.0003	NS	NS
Average area of a Peyer’s patch (mm^2^)	NBW	0.12 ± 0.042 ^a^	0.266 ± 0.032 ^b^	N/A	0.238 ± 0.077 ^a^	0.381 ± 0.03 ^b^	0.59 ± 0.19 ^c^
	IUGR	0.105 ± 0.025	0.174 ± 0.08	N/A	0.086 ± 0.013 ^a^	0.34 ± 0.102 ^b^	0.64 ± 0.13 ^c^
	*p-*Value	0.001	0.0464		0.0001	NS	NS
Total area of Peyer’s patches per section (mm^2^)	NBW	1.67 ± 0.67	4.24 ± 3.51	N/A	5.21 ± 1.92 ^a^	9.71 ± 2.44 ^b^	19.9 ± 10.66 ^c^
	IUGR	1.54 ± 1.14	3.73 ± 2.15	N/A	1.56 ± 0.63 ^a^	8.13 ± 3.25 ^b^	20.0 ± 4.52 ^c^
	*p-*Value	NS	NS		0.0006	NS	NS
Average density of cells per Peyer’s patch (number of cells/mm^2^)	NBW	13,302 ± 2122	7240 ± 2742	N/A	5578 ± 1241 ^a^	5006 ± 1697 ^a^	10362 ± 2498 ^b^
	IUGR	12,177 ± 4582	9379 ± 938	N/A	7263 ± 1205 ^a^	4628 ± 1443 ^b^	8245 ± 2258 ^a^
	*p-*Value	NS	NS		0.02	NS	NS

The *p*-values for IUGR and NBW pairs were obtained using *t*-tests; NS—not significant; ^a,b,c^ different letters in superscript indicate significant differences in each row (*p* < 0.05; *t*-test or one-way ANOVA followed by Tukey’s post-hoc test where appropriate); N/A—not analysed.

**Table 6:** The kinetics of the gut’s immune system development in intrauterine growth restricted (IUGR) pigs from birth to adulthood—a summary.

	PD 0–1	PD 7–8	PD 14	PD 35 *	PD 42	PD 180
Foetal-type enterocytes	ND	**Duo ↑** **Prox ↑↑** **Mid ↑↑** **Ile ↑↑**	**Mid ↑** **Ile ↑↑↑**	N/A	ND	**ND**
Intraepithelial leukocytes	Ile↓	**Duo ↑↑↑** **Pox ↑** **Mid ↑↑↑**	**Prox ↑↑** **Mid ↑**	N/A	N/A	**ND**
Goblet cells	Mid ↓ Ile ↓	**ND**	**Duo ↓↓** **Prox ↓↓** **Mid ↓**	ILE↓	N/A	**ND**
Peyer’s patches	↓	**↓**	**ND**	N/A	N/A	**ND**
Inflammatory cytokines	TNF-α ↓↓ INF-γ ↓↓ IL-10 ↓↓	N/A	TNFα ↑↑	TNF-α ↑↑ INF-γ ↑↑ IL1 ↑↑ IL-6 ↑	INF-γ ↓ IL-1β ↓ IL-6 ↓	N/A
References	[4,9,13]	[13]	[13]	[18]	[33]	

Duo—duodenum; Prox—proximal part of jejunum; Mid—middle part of jejunum; Ile—ileum; PD-postnatal day; *—one week after weaning. Arrows show the directions (↑—increase; ↓—decrease) and intensity of changes (↑—weak, ↑↑↑—strong), as compared to normal pigs. Results in bold are from the present study.

## Data Availability

The data presented in this study are available on request from the corresponding author.
